# Identification of *Schistosoma mansoni* miracidia attractant candidates in infected *Biomphalaria glabrata* using behaviour-guided comparative proteomics

**DOI:** 10.3389/fimmu.2022.954282

**Published:** 2022-10-10

**Authors:** Conor E. Fogarty, Phong Phan, Mary G. Duke, Donald P. McManus, Russell C. Wyeth, Scott F. Cummins, Tianfang Wang

**Affiliations:** ^1^ Centre for Bioinnovation, University of the Sunshine Coast, Maroochydore, QL, Australia; ^2^ School of Science, Technology and Engineering, University of the Sunshine Coast, Maroochydore, QL, Australia; ^3^ Infection and Inflammation Program, Queensland Institute of Medical Research (QIMR) Berghofer Medical Research Institute, Brisbane, QL, Australia; ^4^ Department of Biology, St. Francis Xavier University, Antigonish, NS, Canada

**Keywords:** *Biomphalaria glabrata*, *Schistosoma mansoni*, attractant candidates, behavioural analysis, proteomic comparison

## Abstract

Schistosomiasis, caused by infection with *Schistosoma* digenetic trematodes, is one of the deadliest neglected tropical diseases in the world. The *Schistosoma* lifecycle involves the miracidial infection of an intermediate freshwater snail host, such as *Biomphalaria glabrata*. Dispersing snail host-derived *Schistosoma* miracidia attractants has been considered a method of minimising intermediate host infections and, by extension, human schistosomiasis. The attractiveness of *B. glabrata* to miracidia is known to be reduced following infection; however, the relationship between duration of infection and attractiveness is unclear. Excretory-secretory proteins (ESPs) most abundant in attractive snail conditioned water (SCW) are key candidates to function as miracidia attractants. This study analysed SCW from *B. glabrata* that were naïve (uninfected) and at different time-points post-miracidia exposure (PME; 16h, 1-week, 2-weeks and 3-weeks PME) to identify candidate ESPs mediating *Schistosoma mansoni* miracidia behaviour change, including aggregation and chemoklinokinesis behaviour (random motion, including slowdown and increased turning rate and magnitude). Miracidia behaviour change was only observed post-addition of naïve and 3W-PME SCW, with other treatments inducing significantly weaker behaviour changes. Therefore, ESPs were considered attractant candidates if they were shared between naïve and 3W-PME SCW (or exclusive to the former), contained a predicted N-terminal signal peptide and displayed low identity (<50%) to known proteins outside of the *Biomphalaria* genus. Using these criteria, a total of 6 ESP attractant candidates were identified, including acetylcholine binding protein-like proteins and uncharacterised proteins. Tissue-specific RNA-seq analysis of the genes encoding these 6 ESPs indicated relatively high gene expression within various *B. glabrata* tissues, including the foot, mantle and kidney. Acetylcholine binding protein-like proteins were highly promising due to their high abundance in naïve and 3W-PME SCW, high specificity to *B. glabrata* and high expression in the ovotestis, from which attractants have been previously identified. In summary, this study used proteomics, guided by behavioural assays, to identify miracidia attractant candidates that should be further investigated as potential biocontrols to disrupt miracidia infection and minimise schistosomiasis.

## Introduction

Schistosomiasis is one of the most socioeconomically consequential neglected tropical diseases in the developing world and is caused by digenetic trematodes of the genus *Schistosoma*. The most common causes of human schistosomiasis are infection with *Schistosoma mansoni*, *Schistosoma haematobium* and *Schistosoma japonicum*, which together comprise over 95% of global human schistosomiasis cases (1). Over 200 million people are estimated to be infected with schistosomes, with endemicity ubiquitous in several Middle Eastern, South American and sub-Saharan African nations (2). Most infections are asymptomatic; however, a substantial minority of those infected suffer serious effects, resulting in an estimated over 200,000 deaths per year due to schistosomiasis (3).

Currently, the most common schistosomiasis management method is mass drug administration with praziquantel, a chemotherapeutic drug boasting high efficacy against all *Schistosoma* spp., few side effects and wide spread use for decades (4, 5). However, praziquantel mass drug administration does not prevent re-infections and the drug is less effective against immature schistosomes (6–8). Therefore, other methods of minimising the spread of schistosomiasis are under investigation. Vaccine development and non-pharmacological methods, such as improving sanitation, education and infrastructure, have been thoroughly investigated (9). However, another promising direction in schistosomiasis control is in preventing schistosomes from infecting their respective hosts through targeted chemosensory biocontrols. Due to the high species-specificity and sensitivity of chemosensory responses, the usage of attractants is an enticing, low-risk, targeted method. Chemosensory interference with aquatic species behaviour has recently been demonstrated on several pests, including cane toad (*Rhinella marina*) larvae (10) and European carp (*Cyprinus carpio*) (11), indicating the efficacy of this approach in aquatic settings.

The *Schistosoma* lifecycle involves the infection of an intermediate molluscan host and a definitive mammalian host. Miracidia (which hatch from eggs released into freshwater from definitive hosts) and cercariae (which escape from infected snails) are non-feeding stages which infect molluscan and mammalian hosts, respectively. *Schistosoma* cercariae can infect various mammalian species, including ruminants, humans and mice (12, 13). In contrast, miracidia can only infect snail hosts of a specific genus, such as *Biomphalaria* in the case of *S. mansoni* miracidia. The most widely studied miracidia-host interaction is that between *S. mansoni* miracidia and *Biomphalaria glabrata*, both of which have complete genome databases (14–16). It has been observed that proximity to *B. glabrata* snail-conditioned water (SCW) induces miracidia behaviour change, including chemoklinokinesis (random movement in proximity to a chemical, including slowdown and turning) and aggregation (increased quantity of miracidia presence around the SCW). Chemoklinokinesis has been observed in miracidia of many digenetic trematodes (17, 18). Miracidia of some *S. mansoni* strains display high host specificity, such as Egyptian strain *S. mansoni* for sympatric *Biomphalaria alexandrina* over allopatric *B. glabrata*, indicating preference for sympatric hosts (19). This suggests that miracidia receptors have evolved to identify *Biomphalaria*-specific ligands released into the water. Recent SCW analyses demonstrated that snail-derived peptides and excretory-secretory proteins (ESPs) were the primary stimulants for miracidia behaviour change, while small molecules were less likely to be attractants (20). One such attractant peptide is P12 (—R-*DITSGLDPEVADD*-KR—), which is highly expressed in the central nervous system, foot, heart and kidney and produced identical changes in miracidia behaviour to naïve (uninfected) *B. glabrata* SCW (20). However, aside from P12, few attraction chemicals have been identified (21). Comparative analyses of the chemical composition of attractive and non-attractive SCW may enable the identification of *S. mansoni* miracidia attractants in the former.

In addition to displaying a strong preference for *Biomphalaria*, *S. mansoni* miracidia also display a stronger preference for SCW from naïve *B. glabrata* than SCW from infected *B. glabrata*. When placed in a T-maze, *S. mansoni* miracidia show a significant preference for naïve SCW, but not infected SCW, over an empty chamber (22). This may be due to infected *B. glabrata* releasing fewer attractant ESPs or *S. mansoni* sporocysts (the asexually reproductive stages which miracidia transform into post-infection) releasing deterrent chemicals. Regardless of the mechanism, *S. mansoni* miracidia have an evolutionary justification to avoid infected molluscan hosts; excessive infection of a molluscan host leads to poorer cercarial output. For example, it has been observed that *Galba truncatula* release fewer cercariae when infected with five *Fasciola hepatica* miracidia than when infected with one miracidium (23). Therefore, because infected snails are known to be less attractive to miracidia, comparing SCW from naïve and infected snails may inform the identification of attractant chemicals in the former.

There are some indications that miracidia attraction to snail hosts varies with the duration of host infection. A recent study observed that SCW from *B. glabrata* 3 weeks post-miracidia exposure (PME) induced miracidia aggregation (24). This suggested that attractants may still be present post-infection. Testing SCW from *B. glabrata* at different time-points PME would reveal the relationship between infection duration and attractiveness. If miracidia behaviour change varies substantially between SCW from different time-points PME, a comparative proteomic analysis of ESPs from *B. glabrata* SCW at different time-points post-infection could facilitate the discovery of novel miracidia attractant candidates.

In this study, we aimed to compare SCW collected from *B. glabrata* at different time-points post-exposure to *S. mansoni* miracidia to identify attractant candidates. Firstly, behavioural bioassays were conducted on *S. mansoni* miracidia following the addition of *B. glabrata* SCW from 16h-PME, 1-week PME (1W-PME) and 2W-PME, with 3W-PME and naïve SCW derived from an earlier study (24). Secondly, the ESPs were subjected to semi-quantitative proteomic analysis using LC-MS/MS to identify attractant candidates. Finally, the attractant candidates were analysed using the *B. glabrata* transcriptome to observe their relative expression in different tissue. The findings of this study elucidated the effect of host infection duration on *S. mansoni* miracidia attractiveness and identified several attractant candidates.

## Materials and Methods

### *Biomphalaria glabrata* and Swiss mice maintenance conditions and ethics guidelines

The conduct and procedures involving animal experimentation were approved by the Animal Ethics Committee of the QIMR Berghofer Medical Research Institute, Brisbane (project number P3705). This study was performed in accordance with the recommendations in the Guide for the Care and Use of Laboratory Animals of the National Institutes of Health. The Swiss mice used in this study were subject to Biosecurity Quarantine Control and hence held in a quarantine containment area within a Specific Pathogen Free Animal Facility. The *S. mansoni* were maintained with an Australian Department of Agriculture, Fisheries and Forestry Biosecurity permit. The *B. glabrata* snails (NMRI strain) were maintained in an aerated tank of calcium carbonate conditioned-water (pH-neutral) at 27°C in a 12h alternating cycle of light and darkness. Their diet consisted of algae tablets and lettuce which was washed thoroughly with reverse osmosis water to prevent the introduction of dirt or other undesirable particles.

### Snail-conditioned water collection and semi-purification

Samples of SCW were collected from *B. glabrata* that were naïve and 16h-PME, 1W-PME, 2W-PME and 3W-PME. These time-points were chosen, and each SCW treatment was extracted from a different batch, because frequent SCW collection increases snail stress and accelerates death. No further SCW treatments were collected after 3W-PME due to the high rate of cercarial release after this time-point causing high stress and low SCW protein quantities. The *B. glabrata* snails (60 for each treatment) were washed four times with freshly prepared calcium carbonate-conditioned Milli-Q water to remove any contaminants from the tank and separated into three 50 mL beakers, each containing 20 snails. Snails were incubated in 20 mL of pH-neutral spring water at room temperature for 2 h. Snails were removed and returned to the aquarium, 20 mL of methanol was added to the water samples and mixed thoroughly. The mixture was filtered through a 0.45 µm Durapore PVDF filter (Bedford, MA, USA) to remove contaminants. Filtered samples were immediately frozen on dry ice until lyophilisation using a Savant SpeedVac Concentrator (Thermo Scientific, MA, USA).

### *Schistosoma mansoni* miracidia isolation and behavioural bioassay

Swiss mice infected with *S. mansoni* were euthanised with CO_2_ gas and their livers were finely sliced and placed in room-temperature phosphate-buffered saline (PBS) to collect the eggs of *S. mansoni*. Two sliced livers were shaken to a smooth consistency in 50 mL PBS. The mixture was centrifuged at 2,000×g for 10 s at 20˚C, the supernatant was removed and pellet re-suspended in 50 mL of room temperature PBS. This step was repeated three times until the supernatant was transparent. The eggs were divided between three 50 mL tubes in pH-neutral water covered by alfoil under a light for 2h at room temperature to maximise the quantity of miracidia attained. A total of 2h were allowed for hatching because the infected livers were stored at 4°C the night before being sliced and the eggs were not purified with any further steps (such as using collagenase or filters) (25). The top 4 mL of the water was collected every 30 min for 2 h and the number of miracidia were counted under a microscope. The miracidia were concentrated through centrifuging the water at 4,000×g for 15 min at 20°C and the supernatant was removed. The miracidia were resuspended in 10 mL of Milli-Q water, vortexed and 30 miracidia per 100 µL were counted. This method of SCW behavioural bioassay has been described in detail elsewhere (20).

Briefly, miracidia water aliquots in 100 µL volumes were placed on a hydrophilic glass slide (StarFrost^®^ superclean, hydrophilic slides, ground edges 90°, white, ProSciTech Pty Ltd) to ensure the miracidia were consistently clear and monitored using an Olympus-CKX41 microscope (Olympus) equipped with an Olympus DPI Digital Microscope Camera DP22 (15 frames per second at 2.8-megapixel image quality). Miracidia baseline behaviours were recorded for one minute, followed by addition of 2 µL of SCW, after which a further minute of behaviours was recorded. This process was conducted with nine replicates of *B. glabrata* SCW at 16h-PME, 1W-PME and 2W-PME resuspended in 100 µL of Milli-Q water.

Data for the positive control (naïve SCW), negative control (Milli-Q water) and 3W-PME SCW were obtained in nine replicates from videos recorded in an earlier study (24). There were some minor methodological differences in that study, such as the use of 200 µL aliquots on petri dishes instead of 100 µL aliquots on glass slides, concentrating miracidia at 5,000×g instead of 4,000×g and using 25 snails instead of 20 for behavioural bioassay SCW collection. Behaviour bioassays using Milli-Q and naïve SCW were conducted again using the updated parameters and the new data was compared to the data from the earlier study to observe if the changes in parameters affected miracidia behaviour change. It was calculated that there were no significant differences in any behaviour metrics when comparing different Milli-Q water data and slightly greater decreases in velocity (34% instead of 22%, *P* = 0.0134) and increases in duration of presence (120% instead of 104%, *P* = 0.0448) when comparing naïve SCW data (**Table S1**). Therefore, we concluded that the changes in parameters did not affect behaviour changes enough to alter the conclusions of this paper and hence the naïve SCW, Milli-Q and 3W-PME SCW data were compared to the new PME SCW data.

The videos were analysed using a method described previously (20). Briefly, miracidia were identified when they were within the field of view (FOV). Videos were split into pre-addition and post-addition segments and imported into FIJI software (26). Miracidia contrast was improved using a rolling mean background subtraction method (27). Employing the TrackMate plugin (28), miracidia locations along an x-y axis were tracked in each frame and assembled into complete tracks for each miracidium present in the FOV. Changes were manually performed to prevent miracidia track overlap and eliminate tracks created by remaining liver particles that were unlikely to affect behaviour. The MTrackJ plugin (29) was used to calculate several measures of movement: average velocity (mm/s), angular standard deviation (degrees; measures the standard deviation in the change in angles between consecutive points, therefore reflecting the magnitude and frequency of turns), duration of presence (seconds) and miracidia tracks per min. Behaviour changes of interest included aggregation, signified by an increased quantity of miracidia per min, and chemoklinokinesis, indicated by decreased average velocity and increased duration of presence (both indicating slowdown) and increased angular standard deviation (SD; indicating magnitude and frequency of angle change) between pre-addition and post-addition videos.

For statistical analysis, we used an align-and-rank transform (ART) implemented in the ARTool R package to conduct a nonparametric two-way ANOVA with SCW treatments as a between-subjects factor and pre/post-addition as a within-subjects factor (30). This method avoids the requirement for a normally distributed response variable and accommodates the repeated measures nature of our pre-addition versus post-addition comparisons. This was followed by *post-hoc* contrasts of the changes between pre and post SCW addition for all SCW treatments against Milli-Q water, in addition to the changes between pre and post SCW addition for all PME SCW treatments against naïve SCW (31). A change of *P <* 0.05 was considered significant. Statistical analysis and figure preparation were both performed using R version 4.1.3 with R Studio (32), and the following packages: readxl version 1.4.0 (33), tidyverse version 1.3.1 (34), magrittr version 2.0.3 (35), forcats version 0.5.1 (36), lme4 version 1.1-29 (37), AICcmodavg version 2.3-1 (38), car version 3.1-0 (39), multcomp version 1.4-19 (40), ggplot2 version 3.3.6 (41), plotrix version 3.8-2 (42), ARTool version 0.11.1 (30) and UpSetR version 1.4.0 (43). On the generated box-plot, points beyond 1.5x the interquartile range either below the lower quartile or above the upper quartile are indicated as outliers.

### In-solution trypsin digestion for SCW and sample preparation for LC-MS/MS

While previous studies have identified attractant proteins, such as P12, without in-solution trypsin digestions prior to analysis, preparation typically produced increased result sensitivity (20). Protein concentrations in all *B. glabrata* SCW triplicates, naïve and all time-points PME, were quantified using the BCA (Bicinchoninic Acid) method (44). A total of 25 µg of protein in 100 µL of 6M urea was used from each sample. Aliquots of 1.5 µL of 200 mM dithiothreitol were added to each sample, which were then incubated at 37°C for 1 h. Aliquots of 6 µL of 200 mM iodoacetamide were added to each sample and incubated in the dark at room temperature for 1 h. Volumes of 6 µL of 200 mM dithiothreitol were added to each sample and incubated at room temperature for 45 min. The urea was diluted with 775 µL of Milli-Q water, vortexed, and 20 µL of 100 µg/mL trypsin was added to each sample. The samples were digested at 37°C for 16h. A 15 µL aliquot of 10% formic acid was added to decrease the pH below 3. A Sep-Pak Plus C18 cartridge (Waters) was prepared for each SCW replicate to concentrate the samples. The protocol used was that prescribed for the product (Sep-Pak C18 Plus Short Cartridge, 360 mg Sorbent per Cartridge, 55-105 µm). An 8 mL aliquot of 0.5% acetic acid in 70% acetonitrile, 29.5% Milli-Q water was run through the column and the solution was frozen at -80°C before lyophilisation. Each lyophilised sample was resuspended in 50 µL of 0.1% formic acid and frozen at -20°C until LC-MS/MS analysis.

### uHPLC tandem QTOF MS/MS analyses

SCW treatments were analysed by LC-MS/MS attached to an ExionLC liquid chromatography system (AB SCIEX, Concord, Canada) and a QTOF X500R mass spectrometer (AB SCIEX, Concord, Canada) equipped with an electrospray ion source. A 15 µL aliquot of each *B. glabrata* SCW sample was injected into a 100 mm × 1.7 µm Aeris PEPTIDE XB-C18 100 uHPLC column (Phenomenex, Sydney, Australia) equipped with a SecurityGuard column for mass spectrometry analysis. Linear gradients of 5-35% solvent B over a 10-min period at a flow rate of 400 µL/min, followed by a gradient from 35-80% solvent B over 2 min and 80-95% solvent B in 1 min were used for peptide elution. Solvent B remained at 95% for 1 min to wash the column after which it was decreased to 5% for equilibration prior to the injection of the subsequent sample. Solvent A consisted of 0.1% formic acid in Milli-Q water while solvent B contained 0.1% formic acid in 100% acetonitrile. The ion spray voltage was set to 5500 V, the declustering potential was set to 100 V, the curtain gas flow was set at 30, ion source gas 1 was set at 40, the ion source gas 2 was set at 50 and spray temperature was set at 450^°^C. The mass spectrometer acquired the mass spectral data in an Information Dependant Acquisition mode. Full scan TOFMS data was acquired over the mass range 350-1400 and for product ion ms/ms 50-1800. Ions observed in the TOF-MS scan exceeding a threshold of 100 cps and a charge state of +2 to +5 were set to trigger the acquisition of product ion. The data were acquired and processed using SCIEX OS software (AB SCIEX, Concord, Canada).

### Protein identification

LC-MS/MS data were imported to PEAKS studio (Bioinformatics Solutions Inc., Waterloo, ON, Canada, version 7.0) with the assistance of MSConvert module of ProteoWizard (3.0.1) (45). For the current study, the proteomic data were analysed with the BglaB1.6 database to compare ESPs from different *B. glabrata* SCW treatments (https://vectorbase.org/vectorbase/app/record/dataset/TMPTX_bglaBB02) (15). MS/MS spectra of ESPs were also analysed with reference to the *S. mansoni* database (https://parasite.wormbase.org/Schistosoma_mansoni_prjea36577/Info/Index). *De novo* sequencing of peptides, database searches and characterising specific PTMs (post-translational modifications) were used to analyse the raw data; false discovery rate (FDR) was set to ≤ 1%, and [-10×log(*P*)] was calculated accordingly where *P* was the probability that an observed match was a random event. The PEAKS used the following parameters: (i) precursor ion mass tolerance, 20 ppm; (ii) fragment ion mass tolerance, 0.1 Da (the error tolerance); (iii) tryptic enzyme specificity with two missed cleavages allowed; (iv) monoisostopic precursor mass and fragment ion mass; (v) a fixed modification of cysteine carbamidomethylation; and (vi) variable modifications including lysine acetylation, deamidation on asparagine and glutamine, oxidation of methionine and conversion of glutamic acid and glutamine to pyroglutamate. ESPs were considered to be present at a specific time-point with confidence only when they were present in at least two of the three replicates. This ensures that ESPs are not removed from consideration due to absence from one replicate. The mass spectrometry proteomics data have been deposited to the ProteomeXchange Consortium *via* the PRIDE partner repository with the dataset identifier PXD031989 (46).

### Semi-quantitative protein analysis

Semi-quantitative analysis of ESPs of all *B. glabrata* SCW triplicates, naïve and PME, was carried out using the label-free quantification module PEAKS Q of PEAKS Studio v7.0, based on the relative intensities of featured peptides detected in replicates. The concentrations of extracted ESPs in different replicate samples were measured using BCA method on a NanoDrop 2000c spectrophotometer (Thermo Scientific, Waltham, USA) for the purpose of normalisation. Biological triplicates of each time-point were used in tandem repeats for LC-MS/MS procedure as described above, and the relative concentrations of ESPs were compared and presented as the final results. The mass shift between different runs was set to 20 ppm, and 0.3 min was used for evaluating the retention time shift tolerance. Featured peptides with FDR threshold 1%, including PTMs mentioned above, were included in the quantitative analysis. The result of peptides was first filtrated based on: (i) ratio *versus* quality-score and a fold change of 8 was used; (ii) ratio *versus* average-area (MS signal intensity) set to a fold change of 8; (iii) charge of featured peptides set to between 2 and 5; (iv) fold change of peptide ≥1; and (v) featured peptide detected in more than one sample of the triplicate. Furthermore, protein results were filtered with FDR ≤ 1%, the number of unique peptides ≥1 and fold change ≥1.5. Proteins which did not meet these criteria could not be semi-quantitatively analysed. The abundance of proteins was normalised and compared, and proteins were clustered using one minus Pearson correlation.

### Prediction of secreted proteins, gene ontology and KEGG pathway analysis

Identified ESPs were subjected to BLASTp using non-redundant protein sequences of NCBI. Protein N-terminal signal peptide sequences were predicted using SignalP 5.0 (47) with the transmembrane domains predicted by TMHMM (48). For SignalP predictions, positive identifications were made when both neural network and hidden Markov model algorithms gave coincident estimations. Herein, a protein was designated as secreted only when it met the criterium of SignalP for containing an N-terminal signal peptide and did not have a transmembrane domain predicted by TMHMM. BLAST results were combined and imported to BLAST2GO (49) (version 5.1), to perform gene ontology (GO) and KEGG pathway analyses. Fisher’s exact test was carried out to evaluate the enrichment of GO terms in SCW ESPs with reference to entire proteome of *B. glabrata* (50). The SCW ESPs were also referenced with respect to the *S. mansoni* proteome. The significant GO terms with *P <* 0.01 were considered as over-represented, and FDRs were calculated from *P*-values using the Benjamini-Hochberg procedure (51).

### Identification and analysis of *Schistosoma* miracidia attractant candidates

ESPs shared between SCW samples that induced miracidia behaviour change (naïve and 3W-PME), including aggregation and chemoklinokinesis, were initially identified as miracidia attractant candidates. Therefore, ESPs shared between 3W-PME SCW and naïve SCW analysed following an in-gel digestion, reported previously by Fogarty et al. (24), were also considered. Another essential criterion for consideration as an attractant candidate was high specificity to the *Biomphalaria* genus (identity percentage cut-off of below 50% in species outside the *Biomphalaria* genus according to BLASTp using non-redundant protein sequences of NCBI). Further proteomic analyses were performed to assign cellular function, including using Pfam (52), Panther (53) and InterProScan (54). Phylogenetic analyses used protein sequence alignments generated using MEGA X software (version 10.1.8) (34) with parameters set as follows: algorithm, ClustalW; gap opening penalty, 10; gap extension penalty, 0.2. Visualisation of alignments was carried out on TeXworks software (55) and edited in Adobe Illustrator. The evolutionary history was inferred using the Maximum Likelihood method based on the Jones-Taylor-Thornton matrix-based model. The tree with the highest log likelihood was shown (56). Initial tree for the heuristic search was obtained automatically by applying Neighor-Joining and BioNJ algorithms to a matrix of pairwise distances estimated using a Jones-Taylor-Thornton model, and then selecting the topology with superior log likelihood. The tree is drawn to scale, with branch lengths measured in the number of substitutions per site. All positions containing gaps and missing data were eliminated.

Predicted structure homology modelling was performed using the SWISS-MODEL workspace (57). In brief, a template search with BLAST and HHblits was performed using the primary amino acid sequence against the SWISS-MODEL template library. An initial HHblits profile was built (58), then models were built based on the target-template alignment using ProMod3 (59). The global and per-residue model quality was assessed using the QMEAN scoring function (60). Furthermore, *B. glabrata* proteins were considered more likely to be attractant candidates if they contained features of a secreted precursor protein (N-terminal signal peptide, no transmembrane domains). *B. glabrata* tissue gene expression data was retrieved from a previous study (15). Gene expression levels of these transcriptomes were calculated by mapping raw sequence data against the *B. glabrata* reference genome (structural version annotation BglaB1.6) derived from Vectorbase (https://vectorbase.org/vectorbase/app/record/dataset/TMPTX_bglaBB02) using CLC Genomic Workbench with default parameters (61). A heatmap was constructed using log base 2 of TPM (transcript per million) of protein precursors following the previously described guideline (62).

## Results

### *Schistosoma mansoni* miracidia behaviour bioassays

**Figure 1:**
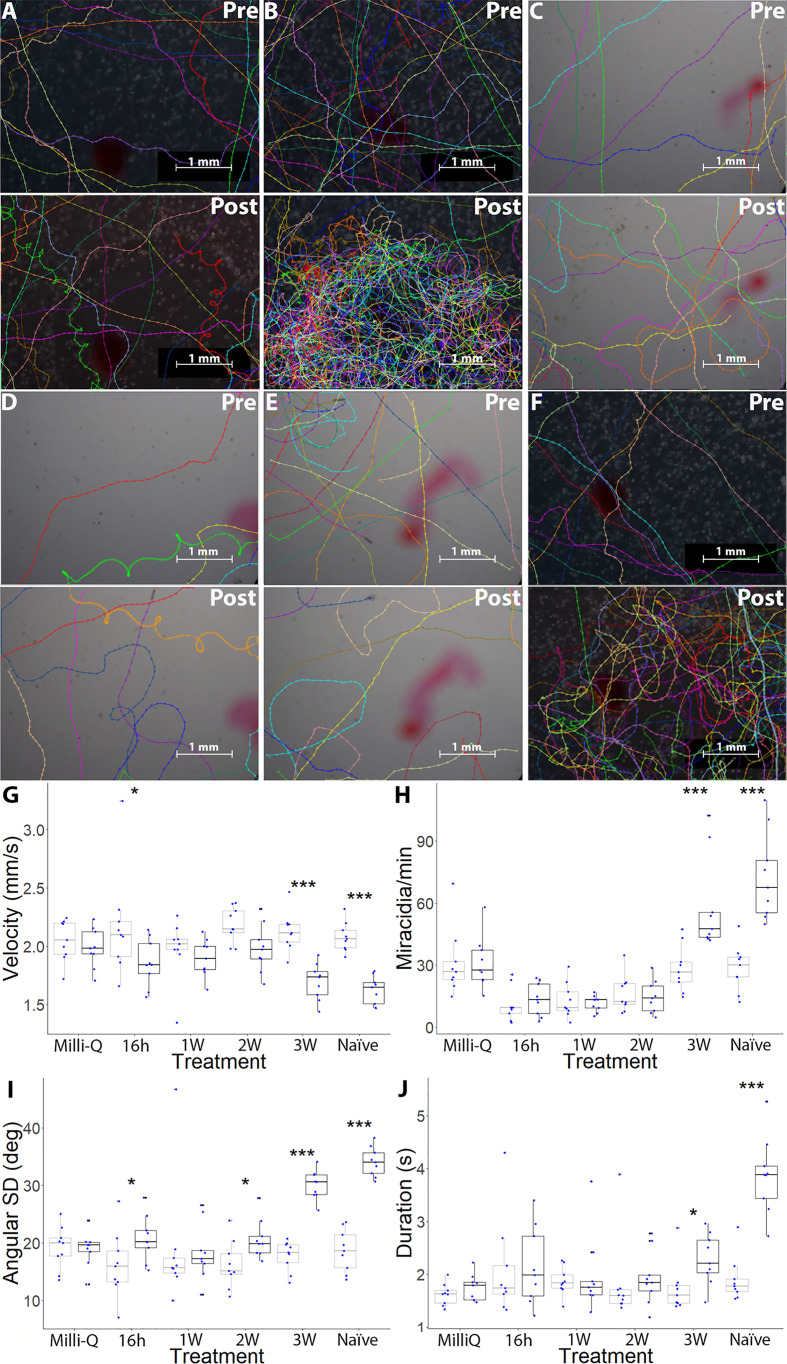
Changes in *S. mansoni* miracidia behaviour from 1 min pre-addition and post-addition of pH-neutral Milli-Q water, 16h-PME, 1W-PME, 2W-PME, 3W-PME and naïve SCW. A representation of miracidia tracks, generated in TrackMate and manually corrected, pre-addition and post-addition of 2 μL of **(A)** Milli-Q water, **(B)** naïve SCW, **(C)** 16h-PME SCW, **(D)** 1W-PME SCW, **(E)** 2W-PME SCW and **(F)** 3W-PME SCW (24). See **Video S1**-**S6** for representative videos. Scale bars are in the bottom right-hand corner of every representative image. Boxplot indicates median, 25^th^ and 75^th^ percentiles, minima and maxima. **(G)** Mean velocity (mm/s); **(H)** average track per min; **(I)** angular SD (degrees) and **(J)** average duration of presence (s). Blue dots indicate the data attained from nine individual replicates of data from each treatment. Points beyond 1.5× the interquartile range either below the lower quartile or above the upper quartile are indicated as outliers. A two-way ANOVA test was used to calculate *P*-values for the mixed-effects interaction of pre-addition and post-addition SCW treatments against Milli-Q water: *P < 0.05, **P < 0.01, ***P < 0.001. Colour: Grey: Pre-addition; Black: Post-addition.

*Schistosoma mansoni* miracidia were exposed to SCW derived from *B. glabrata* at different time-points post-miracidia exposure (16h-PME, 1W-PME and 2W-PME). Data for the control and from the SCW of *B. glabrata* that were naïve and 3W-PME were derived from an earlier paper (24). For each treatment, we tested for miracidia behaviour change, consisting of aggregation (increased quantity of miracidia tracks) and chemoklinokinesis (random movement, signified by decreased average velocity and increased duration of presence and angular SD), in the FOV between pre- and post-addition (**Table S2**). Compared to the negative control (Milli-Q water) (**Figure 1A**; **Video S1**), naïve SCW induced both chemoklinokinesis and aggregation, with expected changes in all behavioural measures (**Figure 1B**): a significant 21.96% decrease in mean velocity (*P <* 0.0001), a significant 154.38% increase in the mean miracidia tracks per min (*P <* 0.0001), a significant 82.64% increase in mean angular SD (*P <* 0.0001) and a significant 104.16% increase in mean duration of presence (*P <* 0.0001) (**Table 1**). These changes indicate aggregation accompanied by random motion (i.e. evidence of attraction) (**Video S2**). In contrast, 16h-PME, 1W-PME and 2W-PME SCW only induced minor or insignificant chemoklinokinesis and no significant aggregation (**Table 1**). 16h-PME SCW exposure significantly increased mean angular SD by 28.55% (*P =* 0.0275) and significantly decreased mean velocity by 14.30% (*P =* 0.0358) (**Figure 1C**; **Video S3**). 1W-PME SCW induced the weakest behaviour change, causing no significant differences in any behaviour metrics relative to the control (**Figure 1D** **;** **Video S4**). 2W-PME SCW exposure only induced a significant increase in mean angular SD by 27.57% (*P =* 0.0480) (**Figure 1E** **;** **Video S5**). These behaviour changes were significantly less extreme than those induced by naïve SCW, including the decrease in velocity induced by 16h-PME SCW (*P =* 0.0045) and increase in angular SD induced by both 16h-PME and 2W-PME SCW (*P ≤* 0.0001).

**Table 1:** Two-way ART ANOVA interaction effects, testing changes in each of the behaviour measurements pre vs post-addition of SCW.

Metric	Interaction F	df	P-value	Contrasts (pre-post)	Milli-Q	16h	1W	2W	3W	Naïve
Mean velocity (mm/s)	9.04	5,48	**<0.0001**	vs Milli-Q	–	**0.0358**	0.7458	0.0833	**<0.0001**	**<0.0001**
				vs Naïve	**<0.0001**	**0.0045**	**<0.0001**	**0.0015**	0.5492	–
Tracks per min	27.59	5,48	**<0.0001**	vs Milli-Q	–	0.9541	0.2731	0.3074	**<0.0001**	**<0.0001**
				vs Naïve	**<0.0001**	**<0.0001**	**<0.0001**	**<0.0001**	**0.0129**	–
Angular SD (degrees)	13.39	5,48	**<0.0001**	vs Milli-Q	–	**0.0275**	0.3493	**0.0480**	**<0.0001**	**<0.0001**
				vs Naïve	**<0.0001**	**<0.0001**	**<0.0001**	**<0.0001**	0.3138	–
Average duration of presence (s)	11.35	5,48	**<0.0001**	vs Milli-Q	–	0.4327	0.6003	0.5412	**0.0355**	**<0.0001**
				vs Naïve	**<0.0001**	**<0.0001**	**<0.0001**	**<0.0001**	**0.0004**	–

Measurements: mean velocity (mm/s), average number of tracks per min, angular SD (degrees) and average duration of presence (s). If the interaction effect was significant, pairwise contrasts testing determined whether the difference in behaviour change pre vs post addition was significant between both Milli-Q and each SCW treatment or between naïve SCW and each of the other SCW treatments (SCW collected 16h, 1W, 2W and 3W post-miracidia exposure). Significant data (p-value < 0.05) is in bold.

The addition of 3W-PME SCW produced the greatest increase in aggregation and chemoklinokinesis among miracidia-exposed *B. glabrata* SCW treatments (**Figure 1F** **)**. These behaviour changes included a significant 20.33% decrease in mean velocity (*P <* 0.0001), a significant 105.45% increase in the mean miracidia tracks per min (*P <* 0.0001) a significant 69.37% increase in the mean angular SD (*P <* 0.0001) and a significant 32.01% increase in mean duration of presence (*P =* 0.0355) (**Figures 1G** **–J** **;** **Video S6**). There were no significant differences in the changes in velocity and angular SD between the naïve and 3W-PME SCW; however, naïve SCW induced significantly greater increases in aggregation (*P =* 0.0129) and duration of presence (*P =* 0.0004) than 3W-PME SCW (**Table 1**). This suggests that 3W-PME SCW does not induce identical behaviour change to naïve SCW; however, it nonetheless induced considerably stronger behaviour changes than the earlier time-points PME. Thus, it appeared that *B. glabrata* exposed to *S. mansoni* miracidia had a time-limited reduction in attractiveness to other miracidia. Therefore, attractants were likely most abundant in naïve and 3W-PME SCW. No SCW treatments induced a significant decrease in the quantity or duration of miracidia presence in the FOV, suggesting that there was no significant deterrent behaviour induced by exposure to any treatment.

### *Biomphalaria glabrata* SCW ESP comparative proteomics

**Figure 2:**
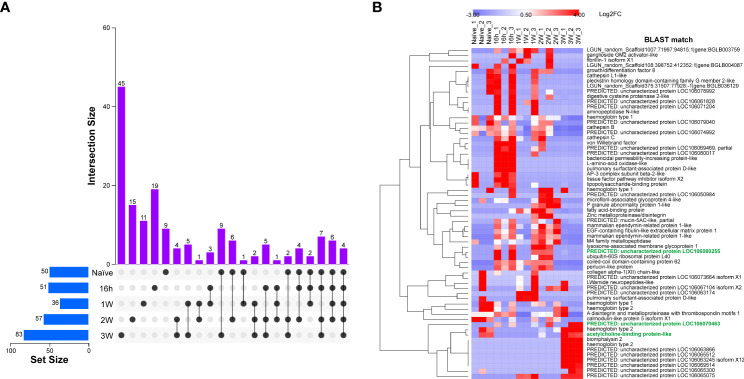
Identification and quantification of ESPs present with confidence from naïve, 16h-PME, 1W-PME, 2W-PME and 3W-PME *B glabrata* SCW. **(A)** An UpSet plot displaying confidence ESP distribution across SCW treatments; **(B)** Heatmap displaying the relative abundance of ESPs identified from SCW triplicates using a label-free semi-quantitative proteomic method. Attractant candidates are denoted with green font.

Triplicates of SCW collected from *B. glabrata*, naïve and at different time-points PME, were analysed by proteomics (**Table S3**). *B. glabrata* ESPs were considered present with confidence when present in at least two of three replicates (**Table S4**). Using this cut-off, a respective total of 50, 51, 36, 57 and 83 confidence *B. glabrata* ESPs were detected in the naïve, 16h-PME, 1W-PME, 2W-PME and 3W-PME SCW treatments (**Figure 2A**). The number of confidence *B. glabrata* ESPs exclusive to naïve, 16h-PME, 1W-PME, 2W-PME and 3W-PME SCW were 9, 19, 11, 15 and 45, respectively (**Table 2**). The greatest overlap in proteins (9 ESPs) occurred between naïve and 3W-PME SCW, while 4 ESPs (M4 family metallopeptidase, uncharacterized protein LOC106074992, mammalian ependymin-related protein 1-like and A disintegrin and metalloproteinase with thrombospondin motifs 1) were shared between all SCW treatments.

**Table 2:** Non-redundant ESPs, identified with confidence, exclusive to *B. glabrata* SCW from snails that were naïve and at 16h-, 1W-, 2W- and 3W-PME to *S. mansoni* miracidia.

Time-point post-miracidia exposure	Accession No.	Description	−10lgP	Coverage (%)^a^	No. of peptides	No. unique^b^	BLASTp e-value
16h	BGLB004262-PB	ganglioside GM2 activator-like	23.68	4	1	1	6.22E-152
	BGLB008634-PB	AP-3 complex subunit beta-2-like	18.86	1	1	1	0
	BGLB009288-PB	multiple EGF and TSP domain-containing protein	15.95	2	1	1	0
	BGLB012280-PB	tissue factor pathway inhibitor isoform X2	44.84	32	3	3	2.04E-45
	BGLB016060-PA	bactericidal permeability-increasing protein-like	117.2	23	11	9	0
	BGLB017086-PA	Hypothetical predicted protein	22.59	6	2	2	0
	BGLB021854-PA	CD109 antigen	29.06	2	2	2	0
	BGLB026410-PA	chorion peroxidase-like	42.64	5	2	2	0
	BGLB030199-PA	L-amino acid oxidase-like	19.3	5	1	1	3.56E-122
	BGLB033580-PA	serpin family protein	62.78	12	5	5	0
	BGLB035660-PA	Bsmp protein	38.61	7	2	2	0
	BGLB036153-PA	uncharacterized protein LOC106069469, partial	44.72	11	3	3	2.96E-105
	BGLB037955-PA	golgin subfamily a member 4	17.59	0	1	1	0
	BGLB040188-PA	lipopolysaccharide-binding protein	120.97	31	11	9	5.39E-164
1W	BGLB008299-PB	actin-5C	49.62	5	2	2	0
	BGLB011796-PB	progranulin-like isoform X1	55.8	2	1	1	0
	BGLB011796-PC	fibrillin-1 isoform X1	55.8	2	1	1	0
	BGLB021817-PA	polyubiquitin-B isoform X2	16.08	6	1	1	1.76E-104
	BGLB035643-PA	uncharacterized protein LOC106055144	30.4	8	1	1	7.28E-80
2W	BGLB000141-PA	microfibril-associated glycoprotein 4-like	37.26	7	3	3	0
	BGLB016967-PA	calmodulin-like protein 5 isoform X3	41.59	10	1	1	9.67E-82
	BGLB019617-PA	uncharacterized protein LOC106050984	48.27	11	1	1	1.64E-54
	BGLB031523-PB	uncharacterized protein LOC106067104 isoform X3	69.5	12	2	2	4.40E-137
	BGLB034051-PA	uncharacterized protein LOC106054319	21.21	6	1	1	2.59E-116
	BGLB040281-PA	Zinc metalloproteinase/disintegrin	36.23	3	1	1	0
3W	BGLB000016-PA	biomphalysin 8	24.5	2	1	1	0
	BGLB000033-PA	biomphalysin 2	131.13	17	10	8	0
	BGLB005531-PC	kazrin-like isoform X1	19.44	1	1	1	0
	BGLB014188-PB	serine/threonine-protein phosphatase 6 regulatory ankyrin repeat subunit B-like	17.05	1	1	1	0
	BGLB026487-PA	HEAT repeat domain-containing protein	67.97	6	2	2	0
	BGLB026513-PB	uncharacterized protein LOC106063245 isoform X1	26.91	5	1	1	4.01E-92
	BGLB027975-PA	uncharacterized protein LOC106065300	105.39	22	6	6	1.86E-112
	BGLB028940-PA	uncharacterized protein LOC106063866	105.39	23	6	6	9.07E-129
	BGLB029397-PA	uncharacterized protein LOC106074740	74.38	36	3	3	2.03E-71
	BGLB030954-PA	golgin subfamily B member 1	17.93	3	1	1	0
	BGLB031282-PA	von Willebrand factor d and egf domain-containing protein	16.74	0	1	1	0
	BGLB032561-PA	uncharacterized protein LOC106069515	31.78	10	1	1	3.39E-85
	BGLB035882-PA	uncharacterized protein LOC106054306 (ovipostatin-like)	41.87	7	1	1	8.73E-136
	BGLB036679-PA	uncharacterized protein LOC106077041	17.24	6	1	1	1.91E-72
	BGLB037163-PA	uncharacterized protein LOC106079133	97.22	54	7	7	2.59E-80
	BGLB038355-PA	uncharacterized protein LOC106069514	74.68	16	3	3	3.78E-100
Naïve	BGLB000202-PA	biomphalysin 20	59.12	6	2	2	0
	BGLB001498-PB	calmodulin isoform X2	37.29	12	2	1	5.91E-119
	BGLB005344-PB	beta-glucuronidase isoform X1	32.35	2	1	1	0
	BGLB020170-PA	thioester-containing protein 1	23.22	7	1	1	3.05E-100
	BGLB024127-PA	calmodulin, striated muscle	16.37	6	1	1	9.23E-99
	BGLB030391-PA	probable serine carboxypeptidase CPVL	34.18	3	1	1	0
	BGLB034203-PA	zinc finger ZZ-type and EF-hand domain-containing protein 1-like	15.79	0	1	1	0
	BGLB035135-PA	endothelin-converting enzyme 2-like isoform X3	31.14	2	1	1	0

a The whole protein sequence coverage from the peptides identified with LC-MS/MS.

b The number of identified peptides unique to the protein.

Details of coverage, peptide match number and BLAST confidence (e-value) are shown.

A semi-quantitative analysis was performed to determine the relative abundance of ESPs within different SCW treatments (**Table S5**). Proteins abundant in at least two replicates of naïve SCW included cathepsin B, haemoglobin type 1 and 2, calmodulin-like protein 5 and acetylcholine-binding protein-like (AChBP-like) protein (**Figure 2B**). Abundant ESPs in 3W-PME SCW included haemoglobins type 1 and 2, biomphalysin 2, AChBP-like proteins and several uncharacterised proteins. Some ESPs were highly abundant in both 3W-PME and naïve SCW and relatively low in abundance in all other SCW treatments, including haemoglobin and AChBP-like proteins. Gene ontology analysis was conducted on *B. glabrata* SCW ESPs to investigate differences between SCW treatments (**Figure S1**). The only function exclusively enriched in both naïve and 3W-PME SCW was obsolete pathogenesis (GO:0009405). This describes the process of one organism inflicting harm on another, and its enrichment was entirely due to the exclusivity of biomphalysin to these SCW treatments.

The SCW ESPs were also referenced against the *S. mansoni* protein database. A total of 10 ESPs were identified with confidence across all SCW treatments, 7 of which were from 16h-PME SCW (**Table S4**). However, these ESPs were predominantly ubiquitous, including tubulin alpha-1A chain, polyubiquitin-B, polyubiquitin-C and calmodulin, and thus are unlikely to function as semiochemicals (attractant or deterrent) due to their lack of species specificity.

### Identification and analysis of *Biomphalaria glabrata* miracidia attractant candidates

Snail-conditioned water derived from naïve and 3W-PME *B. glabrata* both induced significant aggregation and chemoklinokinetic behaviour in *S. mansoni* miracidia. Therefore, ESPs were considered more likely to be attractant candidates if shared between naïve and 3W-PME *B. glabrata* SCW, or specific to the latter. To provide a more robust comparative dataset, 3W-PME SCW ESPs shared with naïve SCW from both this study and an in-gel digestion from an earlier study (24) were considered. Of the 161 proteins identified with confidence in this paper, 79 were shared with naïve SCW in the earlier paper. Of these, only 9, 1, 13 and 5 ESPs were shared with ESPs specific to 16h-PME, 1W-PME, 2W-PME and 3W-PME SCW, respectively, supporting that differences in ESPs are primarily a result of infection instead of the method of analysis. A total of 18 non-redundant proteins met this criterion for consideration as attractant candidates, including biomphalysin 2 and 20, two AChBP-like proteins, mucin-5AC-like protein, calmodulin-like protein 5 isoform X1, haemoglobin types 1 and 2, thioester-containing protein 1 and 6 uncharacterised proteins (**Table 3**; **Table S6**).

**Table 3:** Attractant candidate ESPs identified as shared between *B. glabrata* 3W-PME and naïve SCW, or unique to the latter.

Attractant candidate	SCW treatment	Accession no.	Description	−10lgP	Coverage (%)^a^	No. peptides	No. unique^b^	BLASTp e-value	Signal peptide	Trans-membrane domain	Identity (%)	Pfam
Y	Naïve, 16h, 2W, 3W	BGLB017354-PA	Uncharacterized protein LOC106070463	95.44	25	5	5	2.62E-162	Y	0	32.69	–
Y	Naïve, 3W	BGLB020983-PA/BGLB020983-PB	Acetylcholine-binding protein-like	37.27	7	1	1	1.3E-164	Y	0	35.71	Ligand-gated ion channel
Y	Naïve, 2W, 3W	BGLB021783-PA	Uncharacterized protein LOC106056935	65.41	6	4	4	0	Y	0	–	–
Y	Naïve, 3W	BGLB025228-PA/BGLB025228-PB	Acetylcholine-binding protein-like	98.17	17	4	4	2.94E-166	Y	0	41.71	Ligand-gated ion channel
Y	Naïve, 16h, 2W, 3W	BGLB029661-PA	Uncharacterized protein LOC106080255	62.86	8	2	2	0	Y	0	38.87	H-type lectin domain
Y	Naïve, 2W, 3W	BGLB031523-PA	Uncharacterized protein LOC106067104 isoform X1	145.85	36	7	7	8.00E-149	Y	0	29.60	–
N	Naïve, 3W	BGLB000033-PB	Biomphalysin 2	131.13	17	10	8	0	Y	0	51.99	Aerolysin
N	Naïve	BGLB000202-PA	Biomphalysin 20	59.12	6	2	2	0	N	0	31.65	Aerolysin
N	Naïve	BGLB010468-PB	Haemoglobin type 2	55.78	25	4	1	1.48E-82	N	0	62.39	Globin
N	Naïve, 3W	BGLB011149-PB	Haemoglobin type 2	38.55	7	2	1	0	N	0	56.68	Globin
N	Naïve, 16h, 2W, 3W	BGLB013891-PB	PREDICTED: mucin-5AC-like, partial	17.13	2	1	1	1.29E-119	N	0	–	–
N	Naïve, 3W	BGLB018373-PA	Haemoglobin type 2	107.9	53	9	6	4.34E-82	N	0	63.30	Globin
N	Naïve, 16h, 2W, 3W	BGLB019194-PA	Haemoglobin type 1	80.57	4	3	3	4.34E-82	Y	TMhelix(7-29), outside (30-783)	67.59	Globin
N	Naïve	BGLB020170-PA	Thioester-containing protein 1	23.22	7	1	1	3.05E-100	N	0	74.31	TED_ complement
N	Naïve, 16h, 1W, 2W, 3W	BGLB027972-PA	Uncharacterized protein LOC106074992	35.43	5	1	1	5.26E-149	N	0	29.48	–
N	Naïve, 3W	BGLB030063-PA	Haemoglobin type 1	68.35	12	2	2	1.81E-138	N	0	69.57	Globin
N	Naïve, 2W, 3W	BGLB033943-PA	Calmodulin-like protein 5 isoform X1	64.1	16	2	2	1.00E-75	Y	0	56.73	EF-hand_5
N	Naïve, 3W	BGLB035882-PA	Uncharacterized protein LOC106054306/Ovipostatin-like	41.87	7	1	1	8.73E-136	Y	0	62.07	–

a The whole protein sequence coverage from the peptides identified with LC-MS/MS.

b The number of identified peptides unique to the protein.

Details include SCW treatment of presence, accession no., description, coverage, peptide match number, BLAST confidence (e-value), predicted signal peptide and transmembrane domain presence, highest identity of proteins outside of *Biomphalaria* and Pfam analysis data. ESPs were designated as attractant candidates if they were predicted to contain a signal peptide and lack a transmembrane domain and have a maximum identity of below 50% outside of *Biomphalaria*.

Due to the specificity in snail host genus preference of *S. mansoni* miracidia, ESPs were considered more likely to function as attractants if they were lacking similar homologues outside the *Biomphalaria* genus (**Table 3**). Additionally, ESPs predicted to lack an N-terminal signal peptide or contain a transmembrane domain were excluded, as they were considered unlikely to be excretory-secretory (**File S1**). A total of 6 ESPs ultimately met all our criteria and therefore represented promising miracidia attractant candidates, including two AChBP-like proteins and uncharacterised proteins LOC106070463, LOC106080255, LOC106056935 and LOC106067104 isoform X1 (**File S2**). Of these, only AChBP-like proteins were exclusive to naïve and 3W-PME SCW. Uncharacterised proteins LOC106056935 and LOC106067104 isoform X1 were also shared with 2W-PME SCW and uncharacterised proteins LOC106070463 and LOC106080255 were present in all SCW treatments except for 1W-PME (**Table 3**). The relative abundance of attractant candidates across the SCW treatment triplicates was also considered. An AChBP-like protein was of highest abundance in at least two replicates of both naïve and 3W-PME SCW, yet absent from all other SCW treatment replicates (see **Figure 2B**). In contrast, uncharacterised protein LOC106070463 had low abundance in naïve SCW, while uncharacterised protein LOC106080255 had low abundance in both naïve and 3W-PME SCW. Relative abundance could not be calculated for uncharacterised protein LOC106056935 or LOC106067104 isoform X1 because these proteins did not meet the criteria for semi-quantitative comparison.

**Figure 3:**
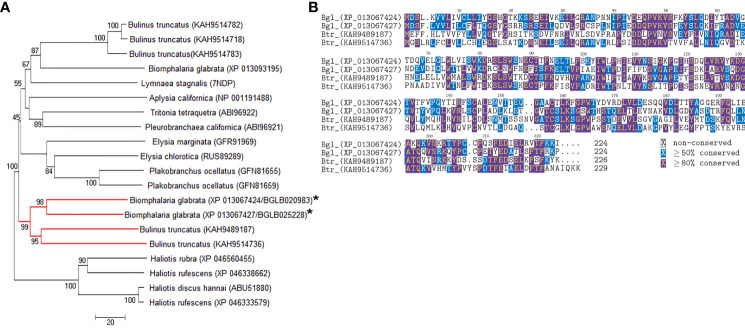
Comparative analysis of AChBP-like proteins in molluscs. **(A)** Phylogenetic analysis with *B glabrata* proteins identified in SCW denoted with asterisk and the red branch demonstrating the clade of interest. **(B)** Protein multiple sequence alignment. Bgl, *B glabrata*; Btr, *B truncatus*.

The AChBP-like proteins and uncharacterised proteins LOC106067104 isoform X1 and LOC106070463 showed some similarity to other known proteins; therefore, they were explored in more depth by comparative sequence analysis. Phylogenetic analysis of the AChBP-like proteins (BGLB020983 and BGLB025228) identified in the *B. glabrata* SCW revealed some similarity with known AChBP-like proteins, yet only high confidence similarity with two *Bulinus truncatus* AChBP-like proteins (KAH9489187 and KAH9514736) (**Figure 3A**). Another *B. glabrata* AChBP-like protein (XP 013093195), not identified in any SCW treatment, displayed closer identity with other gastropod molluscs than with the *B. glabrata* AChBP-like proteins identified in the SCW. Comparative protein sequence analysis of the two *B. glabrata* AChBP-like proteins with the two *B. truncatus* AChBP-like proteins showed most conservation within multiple cysteine residues, as well as specific proline (P), tryptophan (W) and valine (V) residues (**Figure 3B**). Additionally, according to both Pfam and Panther analyses, both AChBP-like proteins were predicted to contain a ligand-gated ion channel, suggesting potential for receptor interaction (**Table S6**).

**Figure 4:**
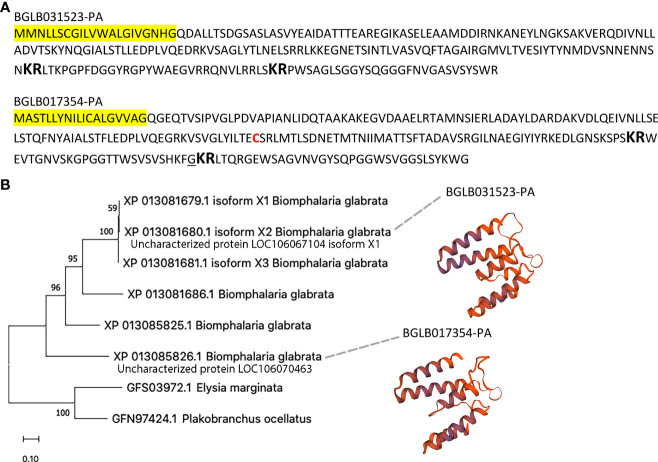
Characterisation of *S. mansoni* attractant candidates uncharacterised proteins LOC106067104 isoform X1 and LOC106070463. **(A)** Annotation of protein sequences, including signal peptide (yellow highlight), dibasic cleavage sites (large bold), cysteine residue (red font) and amidation (underline). **(B)** Phylogenetic analysis and protein models.

Homology was also relatively high between uncharacterised proteins LOC106067104 isoform X1 and LOC106070463 (**Figure 4A**). Despite sharing only 42% overall amino acid identity, several highly conserved regions were identified between the two uncharacterised proteins, including IALSTF/LLEDPLVQED/DRKVSA/AGLY, in addition to two predicted dibasic cleavage sites. Furthermore, they had predicted structural similarity based on predicted protein structure models (**Figure 4B**), in which both exhibited most similar sequence identity (23-28% identity) to a Junction 23, DHR14-DHR18 protein (PDB # 6w2v.2). Four additional sequences with similarity were present in *B. glabrata*, which together show phylogenetic clustering and with only minor identity with two proteins derived from evolutionary distant species (*Elysia marginata* and *Plakobranchus ocellatus*). Uncharacterised protein LOC106070463 displayed similar homology with other *B. glabrata* uncharacterised proteins, including LOC106070462 (49%), LOC106067104 isoform X2 (42%), LOC106067104 isoform X3 (39%) and LOC106067108 (49%). Homology was lower with *Plakobranchus ocellatus* PoB_002393000 (33%) and *Elysia marginata* ElyMa_006483900 (27%), indicating highest conservation within *B. glabrata*.

**Figure 5:**
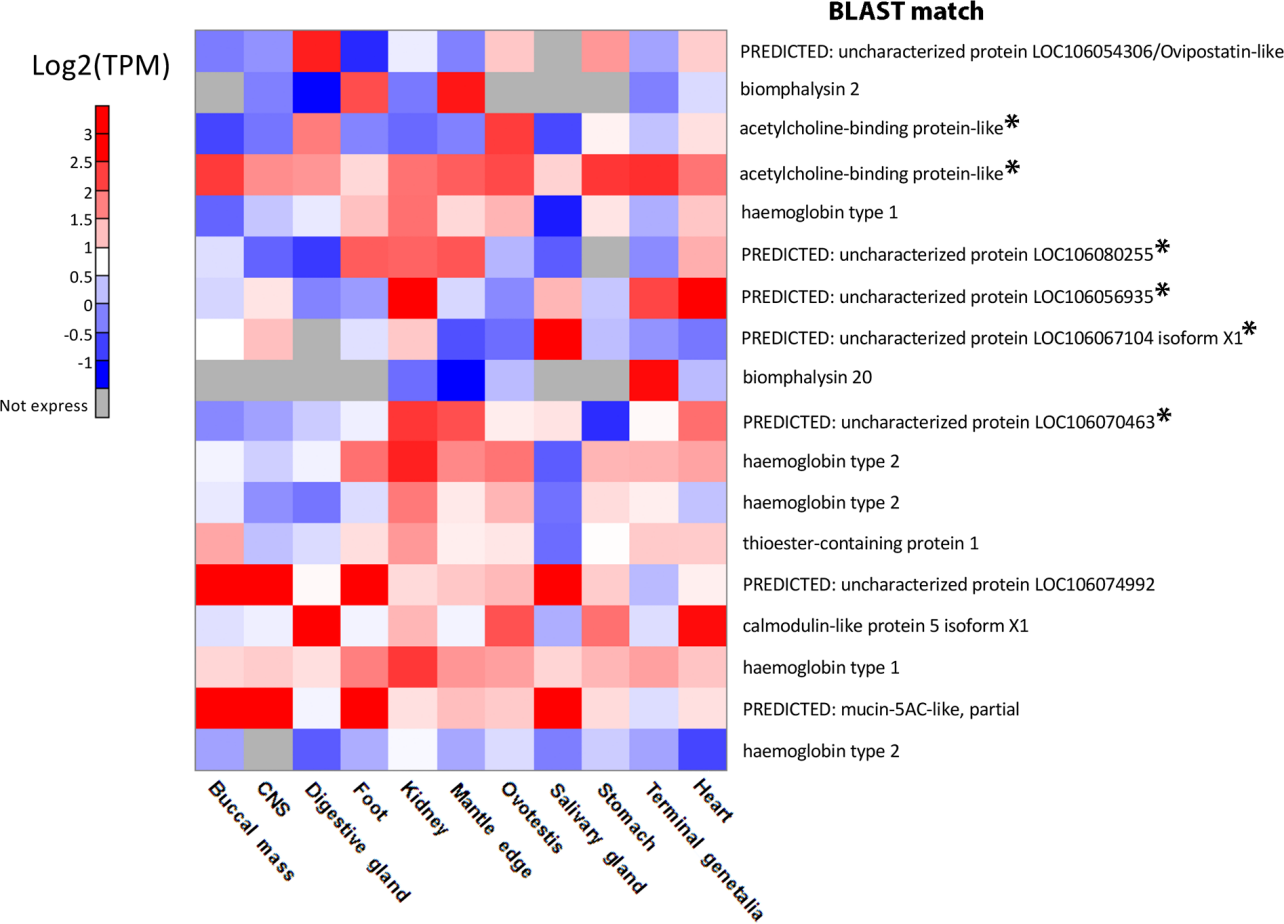
A heatmap displaying the log_2_ gene expression levels of attractant candidates in *B glabrata* tissues. Colour: Red: High expression; Blue: Low expression; Grey: No expression. Attractant candidates have been denoted with an asterisk.

We also investigated the expression of attractant candidate transcripts across different *B. glabrata* tissues using a previous study (15) (**Table S7**). This demonstrated that uncharacterised protein LOC106067104 isoform X1 was most highly upregulated in the salivary gland. Both uncharacterised proteins LOC106080255 and LOC106070463 were relatively highly expressed in the mantle, kidney and heart, with the former also highly expressed in the foot (**Figure 5**). Uncharacterised protein LOC106056935 was also highly expressed in the kidney and heart, in addition to the genitalia and, to a lesser degree, salivary gland. Of the two AChBP-like proteins, one was most highly expressed in the digestive gland and ovotestis and the other was highly expressed in most tissue.

## Discussion

The aim of this study was to comparatively analyse *B. glabrata* SCW at different time-points post-exposure to *S. mansoni* miracidia to identify attractant candidates. This was achieved through comparing the effects of SCW from naïve and 16h-PME, 1W-PME, 2W-PME and 3W-PME *B. glabrata* on miracidia behaviour. Following this, SCW ESPs were analysed using proteomics to identify proteins shared between 3W-PME and naïve SCW, or exclusive to the latter. Attractant candidates were identified based off specificity to *Biomphalaria* and features consistent with being secreted. This facilitated the identification of 6 attractant candidate ESPs with confidence.

### *Schistosoma mansoni* miracidia behaviour bioassay

*Schistosoma mansoni* miracidia behaviour change around *B. glabrata* SCW involves aggregation and chemoklinokinesis (random motion, most commonly increased turning and slowdown) (18, 63, 64). These patterns were observed to varying degrees among the SCW treatments in our study. 3W-PME SCW produced the most similar increases in aggregation and chemoklinokinesis to those induced by naïve SCW. There were no significant differences in changes in miracidia turning or slowdown between 3W-PME and naïve SCW. However, naïve SCW induced significantly greater increases in the quantity and duration of miracidia presence in the FOV. These observations imply that 3W-PME and naïve SCW contain the greatest quantities of attractant ESPs. 16h-PME and 2W-PME SCW both slightly increased turning; however, these increases were significantly smaller than those induced by naïve SCW. This suggests that these SCW treatments likely contained smaller quantities of attractant ESPs. 1W-PME SCW did not produce significant changes in any behavioural metrics, indicating it contained the lowest quantity of SCW attractant(s). The behaviour changes induced by 16h-PME, 1W-PME and 2W-PME SCW can be most accurately characterised as failing to induce behaviour changes associated with attraction, because none of these treatments induced significant aggregation, instead of inducing deterrent behaviour. This is because they induced behaviour changes that were not significantly different to the control, rather than decreasing the quantity or duration of miracidia presence in the FOV. Experiments in search of deterrents should consider exposing miracidia to sporocyst-conditioned water.

It is unknown why SCW from shorter durations PME induced weaker, if any, aggregation and chemoklinokinesis. Because heavily infected *B. glabrata* have drastically shortened lifespans, and thus are sub-optimal hosts, miracidia attraction to 3W-PME SCW was unexpected. It may be speculated that intramolluscan cercariae, germ balls and daughter sporocyst generations do not inhibit the production of miracidia attractants as effectively as mother sporocysts. From these behavioural bioassay results, it may be suspected that the decrease in attractant chemical abundance post-miracidia exposure is only a temporary phenomenon and that infected *B. glabrata* may regain their attractiveness to *S. mansoni* miracidia throughout infection. Future studies should observe SCW from snails after 3W-PME, following the release of potentially hundreds of cercariae, to see if reinfection of snails is more common than was previously believed.

### Attractant candidate identification and analysis

Naïve and 3W-PME *B. glabrata* SCW induced similar behaviour change in *S. mansoni* miracidia. Therefore, ESPs were considered more likely to be attractant candidates if they were specific to naïve SCW or shared with 3W-PME SCW. For consideration as attractant candidates, ESPs were also required to be specific to *Biomphalaria* because ubiquitous proteins are less likely to function as attractants given the parasite’s requirement for species-specificity. This was previously demonstrated by the discovery of the *B. glabrata* attractant peptide, P12, where the protein precursor lacked any known homologues outside of the species (20). Additionally, attractant candidates were required to contain a predicted signal peptide, and lack a transmembrane domain, to confirm that they are excretory-secretory. A total of 6 ESPs met these criteria, including two AChBP-like proteins and uncharacterised proteins LOC106070463, LOC106080255, LOC106056935 and LOC106067104 isoform X1.

Of the attractant candidates identified, AChBP-like proteins appeared of most interest due to their exclusivity and abundance within several replicates of both naïve and 3W-PME SCW. Additionally, they were predicted to contain ligand-gated ion channel domains, suggesting potential to interact with miracidia receptors, including GPCRs and other ligand-gated channels (65). Immunolocalization should be conducted with these proteins to investigate potential miracidia receptor binding capacity. Furthermore, the relatively high expression of one of these proteins in the *B. glabrata* ovotestis may also be noteworthy because *S. mansoni* infections cause chemical castration in infected *Biomphalaria*, a phenomenon which has been well-characterised (66). Ovotestis are among the organs most affected by infection, as evidenced by the significantly decreased concentrations of sex hormones, such as estradiol and testosterone, post-exposure to *S. mansoni* miracidia (67). However, by 4 weeks PME, the concentrations of these hormones in the ovotestis were not significantly different compared to snails prior to infection. Thus, the decreased attractiveness of infected SCW, followed by increased attraction at 3W-PME, appears to loosely correlate with the concentration of these endogenous sex hormones. However, data regarding the role of sex hormones in miracidia attraction is conflicted. A behaviour analysis of miracidia showed that sexual maturity did not significantly impact miracidia preference of SCW or *B. glabrata* over a control (22). Nevertheless, proteins associated with reproduction have been implicated in inducing behaviour change in miracidia. An example is the *B. glabrata* buccalin peptide (21), a miracidia attractant known to play a role in reproduction in snails (and other molluscs) (68, 69). This suggests that sex-related chemicals may be a contributing factor to miracidia attraction; however, the snails likely release several other attractants unrelated to reproduction.

Several additional proteins met our criteria as present in naïve and 3W-PME SCW, but seemed less likely as candidates once further analysis was completed. Uncharacterised proteins LOC106080255 and LOC106070463 were present with confidence in all SCW treatments except for 1W-PME SCW. Furthermore, although present, both proteins were low in abundance in naïve SCW. The encoding genes of both proteins were highly expressed in the foot, kidney and heart, with the encoding gene of LOC106080255 also highly expressed in the mantle. The foot and mantle are common entry points for miracidia and therefore are of interest when identifying attractants. Uncharacterised protein LOC106056935 was only absent from 16h-PME and 1W-PME SCW and its relative abundance could not be calculated. Similar to the aforementioned attractant candidates, its encoding gene was highly expressed in the kidney and heart. Uncharacterised protein LOC106070463 displayed strong homology with uncharacterised protein LOC106067104 isoform X1, which was also present with confidence in naïve, 2W-PME and 3W-PME SCW. However, the abundance of uncharacterised protein LOC106067104 isoform X1 could not be calculated and it was only highly expressed in the salivary gland. Therefore, none of these additional proteins were as promising as attractant candidates as the AChBP-like proteins.

The capacity of 3W-PME and naïve SCW to induce behaviour changes associated with attraction in *S. mansoni* miracidia may be partially explained by the presence of the P12 peptide (20). P12 has several precursor proteins, one of which (uncharacterized protein LOC106065300) was shared exclusively between these two SCW treatments. However, the precursor protein was more consistently abundant in 3W-PME SCW, with high abundance in two replicates of 3W-PME SCW and in only one of naïve SCW. Furthermore, another P12 precursor protein, the uncharacterized protein LOC106063866, was exclusive to 3W-PME SCW and highly abundant in all of its triplicates. The relatively low abundance or absence of these precursors from naïve SCW, despite producing greater behaviour change, may be explained by the presence of other attractant proteins or peptides. AChBP-like proteins are promising as attractant candidates due to their abundance in at least two replicates of both naïve and 3W-PME SCW. Other peptides from the P12 precursor proteins have all previously been discovered and tested; hence, no new attractant candidates could be identified from these precursor proteins (20). Therefore, the presence of other attractants, such as the AChBP-like proteins, are necessary to explain the greater behaviour change induced by naïve SCW despite its lower abundance of P12 precursors.

### Analysis of other SCW ESPs

Outside of the attractant candidates, another protein of interest identified in naïve and 3W-PME SCW was ovipostatin-like protein, an uncharacterised protein highly expressed in the *B. glabrata* ovotestis. While its relative abundance could not be calculated and it was not sufficiently specific to *Biomphalaria* to constitute a species-specific attractant candidate, its exclusivity to naïve and 3W-PME SCW and predicted signal peptide made it of interest. Ovipostatins are a family of proteins originally characterised in *Lymnaea* snails as accessory gland proteins exclusively produced in the prostate (70, 71). It has been identified as highly upregulated during sexual activity, notably 24 hours post-ejaculation (72). Its presence in 3W-PME SCW is consistent with a transcriptomic analysis of *Biomphalaria pfeifferi* post-exposure to miracidia, where ovipostatin 2 was downregulated within one day post-exposure and several days thereafter; however, ovipostatin 5 was upregulated when the snail began to shed *S. mansoni* cercariae. This suggests that later generation sporocysts, germ balls and cercariae do not inhibit the production of ovipostatins as effectively as earlier generation sporocysts. *B. glabrata* ovipostatins, therefore, warrant further investigation to observe their potential effect on miracidia behaviour.

### Conclusions and future directions

This study performed proteomic characterisation on SCW from *B. glabrata*, naïve and at 16h-PME, 1W-PME, 2W-PME and 3W-PME and compared their effect on *S. mansoni* miracidia behaviour. The results indicate that 3W-PME SCW and naïve SCW induce comparable behaviour change, including aggregation and chemoklinokinesis, in miracidia. 16h-PME and 2W-PME SCW caused minor increases in miracidia slowdown and turning, while 1W-PME SCW did not produce any significant changes in behaviour. A total of 6 excretory-secretory attractant candidates were identified as specific to *Biomphalaria* and specific to naïve *B. glabrata* SCW or shared with 3W-PME SCW. This included two AChBP-like proteins and 4 uncharacterised proteins. These attractant candidates should be tested on *S. mansoni* miracidia to observe if they induce behaviour change, as this may disrupt infections and mitigate schistosomiasis.

## Data availability statement

The datasets presented in this study can be found in online repositories. The names of the repository/repositories and accession number(s) can be found below: ProteomeXchange Consortium *via* the PRIDE partner repository with the dataset identifier PXD031989.

## Ethics statement

The conduct and procedures involving animal experimentation were approved by the Animal Ethics Committee of the QIMR Berghofer Medical Research Institute, Brisbane (Project number P3705). This study was performed in accordance with the recommendations in the Guide for the Care and Use of Laboratory Animals of the National Institutes of Health. The Swiss mice used in this study were subject to Biosecurity Quarantine Control and hence held in a quarantine containment area within a Specific Pathogen Free Animal Facility. *S. mansoni* were maintained with an Australian Department Agriculture, Fisheries and Forestry Biosecurity permit.

## Author contributions

CF conceived the study. SC and TW participated in the study design. DM provided the access to snail and parasite materials. CF carried out the experiments and analyzed the data under the supervision of TW and RW. PP performed the transcriptomic comparison. MD maintained the *S. mansoni* miracidia and *B. glabrata* snails and collected samples. CF drafted the manuscript. SC, TW, CF and PP generated the figures. SC, DM, RW and TW provided critical inputs to help draft the manuscript. All authors contributed to the article and approved the submitted version.

## Funding

This study was supported by Australian Research Council Discovery Project (ARC DP180103694). The funders were not involved in the design, data collection and analysis, preparation or publication of the manuscript.

## Acknowledgements

*B. glabrata *snails were provided by the NIAID Schistosomiasis Resource Center of the Biomedical Research Institute (Rockville, MD) through NIH-NIAID Contract HHSN272201700014I for distribution through BEI Resources. We wish to acknowledge QCIF for its support in this research. The authors thank the University of the Sunshine Coast for providing CF a Research Training Program Scholarship and the QIMR Berghofer Medical Research Institute for maintaining the *B. glabrata* snails and *S. mansoni*.

## Conflict of interest

The authors declare that the research was conducted in the absence of any commercial or financial relationships that could be construed as a potential conflict of interest.

## Publisher’s note

All claims expressed in this article are solely those of the authors and do not necessarily represent those of their affiliated organizations, or those of the publisher, the editors and the reviewers. Any product that may be evaluated in this article, or claim that may be made by its manufacturer, is not guaranteed or endorsed by the publisher.

## Supplementary material

Supplementary Figure 1 Gene ontology enrichment analysis of ESPs in SCW of *B. glabrata*, naïve and at 16h-PME, 1W-PME, 2W-PME and 3W-PME. The GO terms enriched in **(A)**: naïve SCW; **(B)**: 16h-PME SCW; **(C)**: 1W-PME SCW; **(D)**: 2W-PME SCW and **(E)**: 3W-PME SCW. The *B. glabrata* genome-derived proteome was used as the reference set in the analysis and P-value was set to below 0.05. Colour: Yellow: Biological Process; Pink: Molecular Function; Purple: Cellular Component.

File S1 SignalP schematic of attractant candidates.

File S2 Annotation of attractant candidates derived from *B. glabrata* SCW, including signal peptide (yellow highlight), dibasic cleavage sites (large bold), cysteine residue (red font) and amidation (underline).

Supplementary Table 1 Behavioural bioassay guided data, including tracks data, from *S. mansoni* miracidia one min pre-addition and post- addition of Milli-Q water and naïve from an earlier study (24) and using the parameters employed for 16h-, 1W- and 2W-PME SCW. A Two-way ART ANOVA interaction effects analysis, testing changes in each of the behaviour measurements pre vs post-addition of SCW. Measurements: mean velocity (mm/s), average duration of presence (s), angular SD (degrees) and average number of tracks per min. If the interaction effect was bolded, pairwise contrasts testing whether the change in behaviour pre vs post addition was different was conducted between Milli-Q and naïve SCW data attained using bioassays with different parameters.

Supplementary Table 2 Behavioural bioassay guided data, including tracks data, from *S. mansoni* miracidia one min pre-addition and post- addition of Milli-Q water and naïve, 16h-PME, 1W-PME, 2W-PME and 3W-PME *B. glabrata* SCW.

Supplementary Table 3 A list of all proteins and corresponding peptides identified in all replicates of 16h-PME, 1W-PME, 2W-PME, 3W-PME and naïve *B. glabrata* SCW with reference to the *B. glabrata* and *S. mansoni* proteomes.

Supplementary Table 4 A list of all proteins identified in at least two replicates of 16h-PME, 1W-PME, 2W-PME, 3W-PME and naïve *B. glabrata* SCW with reference to the *B. glabrata* and *S. mansoni* proteomes.

Supplementary Table 5 Semi-quantitative description of proteins in naïve, 16h-PME, 1W-PME, 2W-PME and 3W-PME *B. glabrata* SCW, including supporting peptides and log_2_ (Fold change).

Supplementary Table 6 A complete description of ESPs identified as shared between 3W-PME and naïve SCW or specific to the latter. This includes attractant candidate status, SCW treatment presence, gene ontology, Pfam and Panther analysis, TMHMM and SignalP results and unique peptides.

Supplementary Table 7 Gene expression levels derived from the *B. glabrata* reference genome (structural version annotation BglaB1.6) derived from Vectorbase (https://vectorbase.org/vectorbase/app/record/dataset/TMPTX_bglaBB02) using CLC Genomic Workbench with default parameters.

Supplementary Video 1 A representative video of *S. mansoni* miracidia one min pre-addition and post- addition of Milli-Q water (Speed ×2, MP4 5.51 Mb). A scale bar is in the bottom right-hand corner of the video.

Supplementary Video 2 A representative video of *S. mansoni* miracidia one min pre-addition and post- addition of naïve *B. glabrata* SCW (Speed ×2, MP4 5.52 Mb). A scale bar is in the bottom right-hand corner of the video.

Supplementary Video 3 A representative video of *S. mansoni* miracidia one min pre-addition and post- addition of 16h-PME *B. glabrata* SCW (Speed ×2, MP4 3.55 Mb). A scale bar is in the bottom right-hand corner of the video.

Supplementary Video 4 A representative video of *S. mansoni* miracidia one min pre-addition and post- addition of 1W-PME *B. glabrata* SCW (Speed ×2, MP4 3.57 Mb). A scale bar is in the bottom right-hand corner of the video.

Supplementary Video 5 A representative video of *S. mansoni* miracidia one min pre-addition and post- addition of 2W-PME *B. glabrata* SCW (Speed ×2, MP4 3.14 Mb). A scale bar is in the bottom right-hand corner of the video.

Supplementary Video 6 A representative video of *S. mansoni* miracidia one min pre-addition and post- addition of 3W-PME *B. glabrata* SCW (Speed ×2, MP4 5.30 Mb). A scale bar is in the bottom right-hand corner of the video.

## Abbreviations

AChBP, Acetylcholine-binding protein; ESP, excretory–secretory protein; FDR, false discovery rate; FOV, field of view; GO, gene ontology; LC-MS/MS, liquid chromatography with tandem mass spectrometry; PBS, phosphate-buffered saline; PME, post-miracidia infection; QIMR, Queensland Institute of Medical Research; SCW, snail-conditioned water.
