# Supplementary figures and images for: Identification of Schistosoma mansoni miracidia attractant candidates in infected Biomphalaria glabrata using behaviour-guided comparative proteomics
Source: Front Immunol. 2022 Oct 10;13:954282. doi: 10.3389/fimmu.2022.954282 (PMC9589101; doi:10.3389/fimmu.2022.954282)

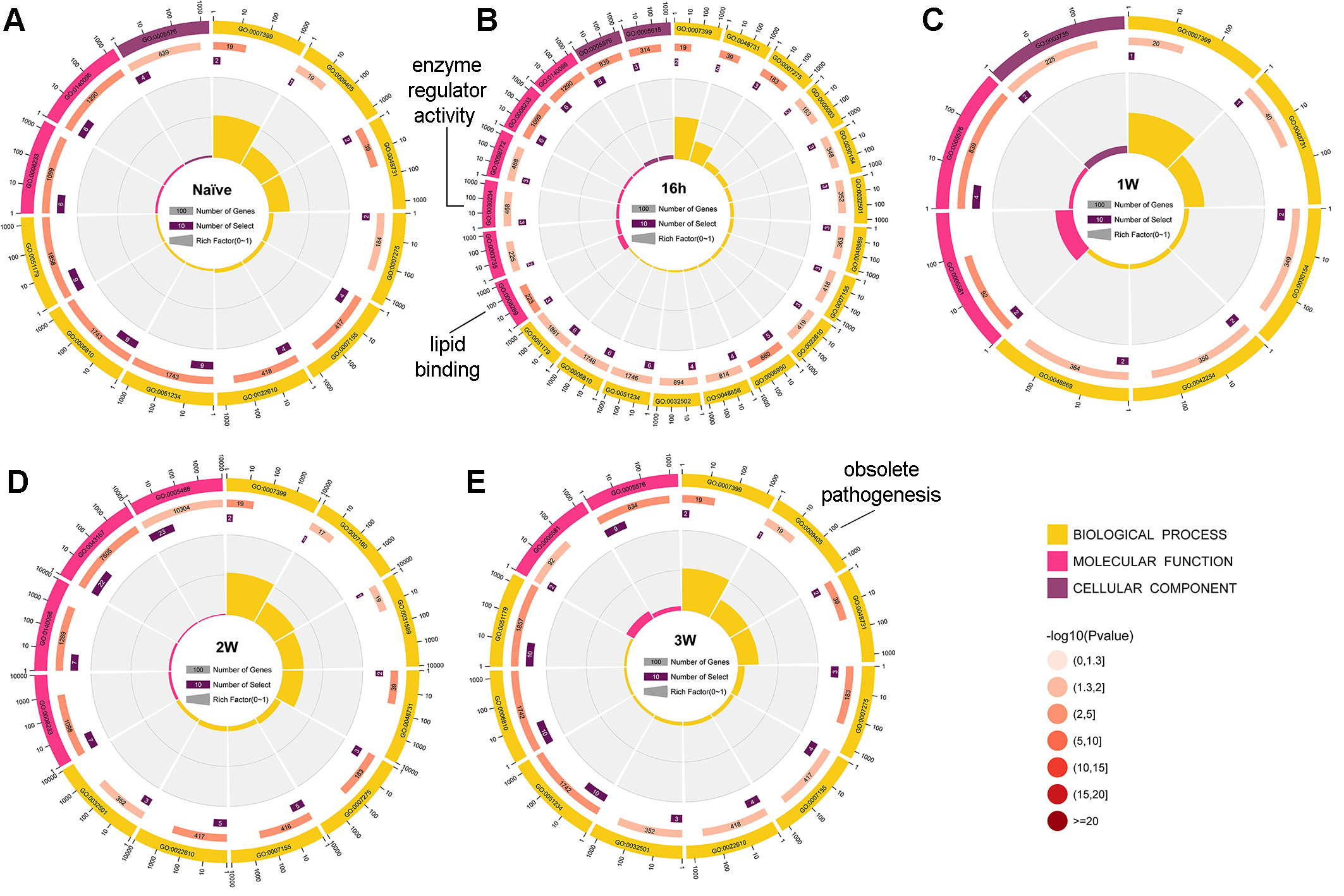

Supplement: Supplementary Figure 1 — Gene ontology enrichment analysis of ESPs in SCW of B. glabrata, naïve and at 16h-PME, 1W-PME, 2W-PME and 3W-PME. The GO terms enriched in (A): naïve SCW; (B): 16h-PME SCW; (C): 1W-PME SCW; (D): 2W-PME SCW and (E): 3W-PME SCW. The B. glabrata genome-derived proteome was used as the reference set in the analysis and P-value was set to below 0.05. Colour: Yellow: Biological Process; Pink: Molecular Function; Purple: Cellular Component. [file Image_1.tif]
