# Supplementary material for: Identification of Schistosoma mansoni miracidia attractant candidates in infected Biomphalaria glabrata using behaviour-guided comparative proteomics
Source: Front Immunol. 2022 Oct 10;13:954282. doi: 10.3389/fimmu.2022.954282 (PMC9589101; doi:10.3389/fimmu.2022.954282)

**File S1.** SignalP schematics of attractant candidates identified in **Table 3**.

Description: Uncharacterized protein LOC106070463


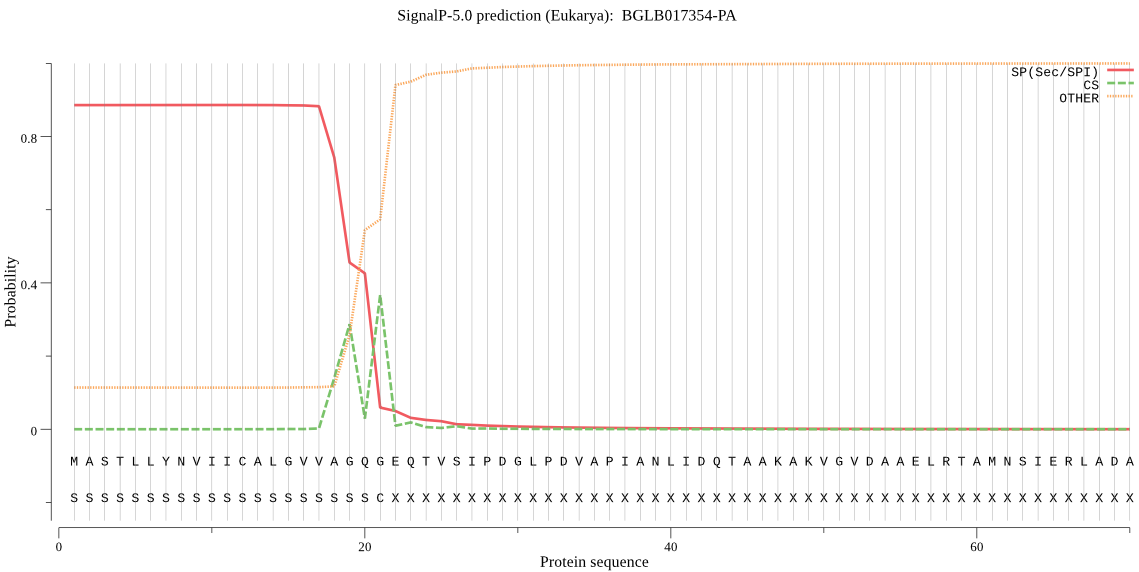


Description: Acetylcholine-binding protein-like
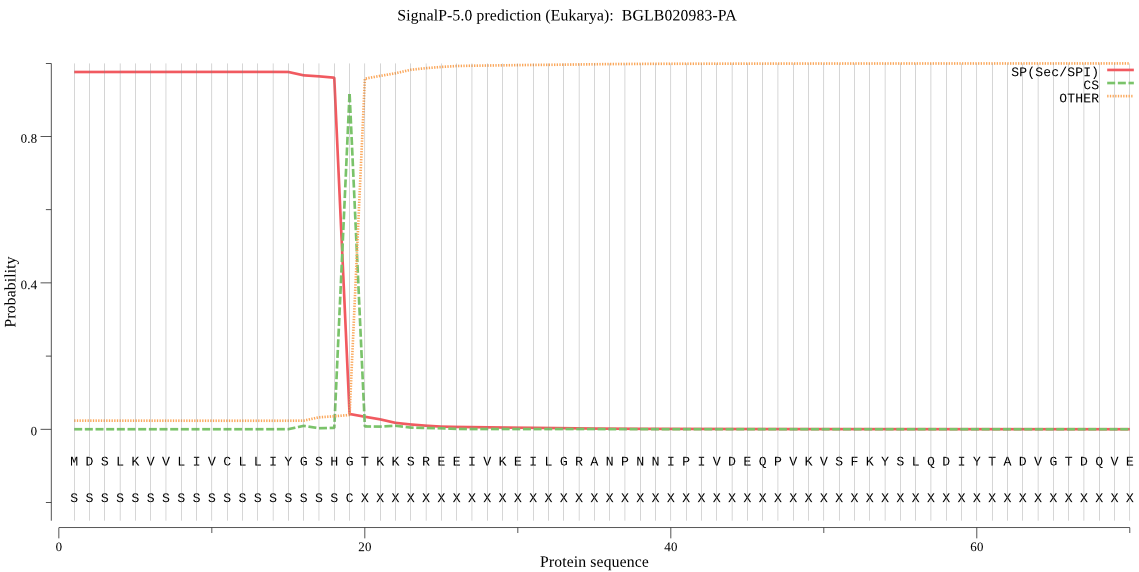


Description: Uncharacterized protein LOC106080255


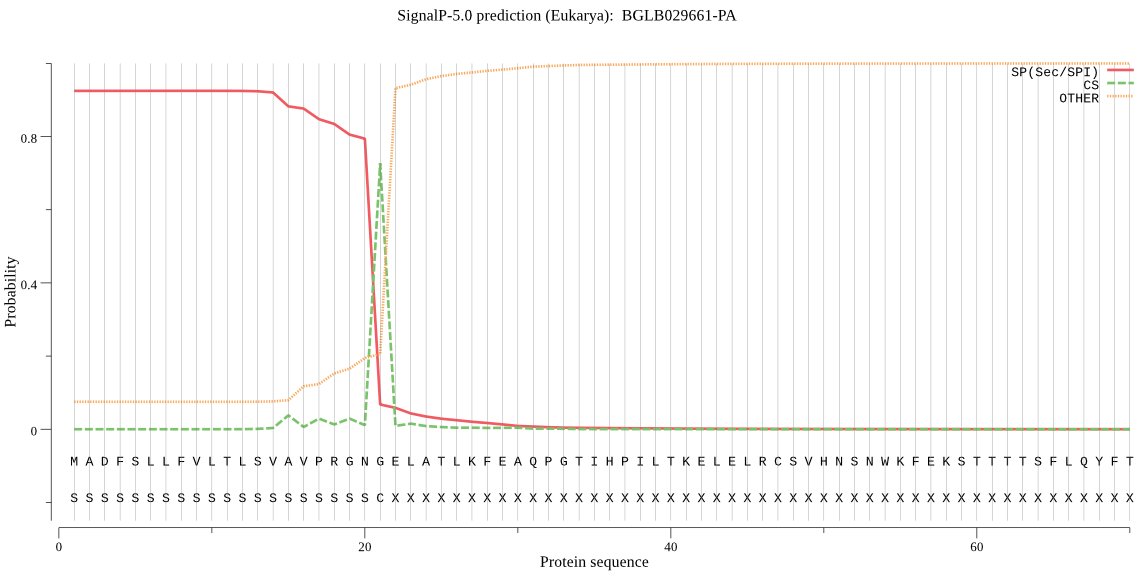


Description: Uncharacterized protein LOC106056935
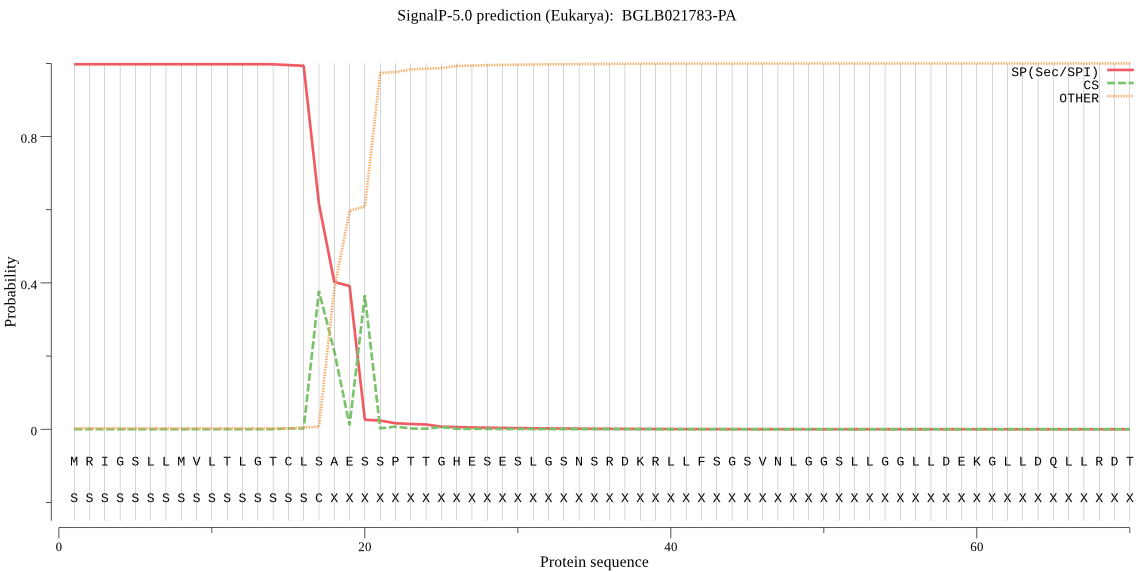


­­­­­

­­

Description: Acetylcholine-binding protein-like
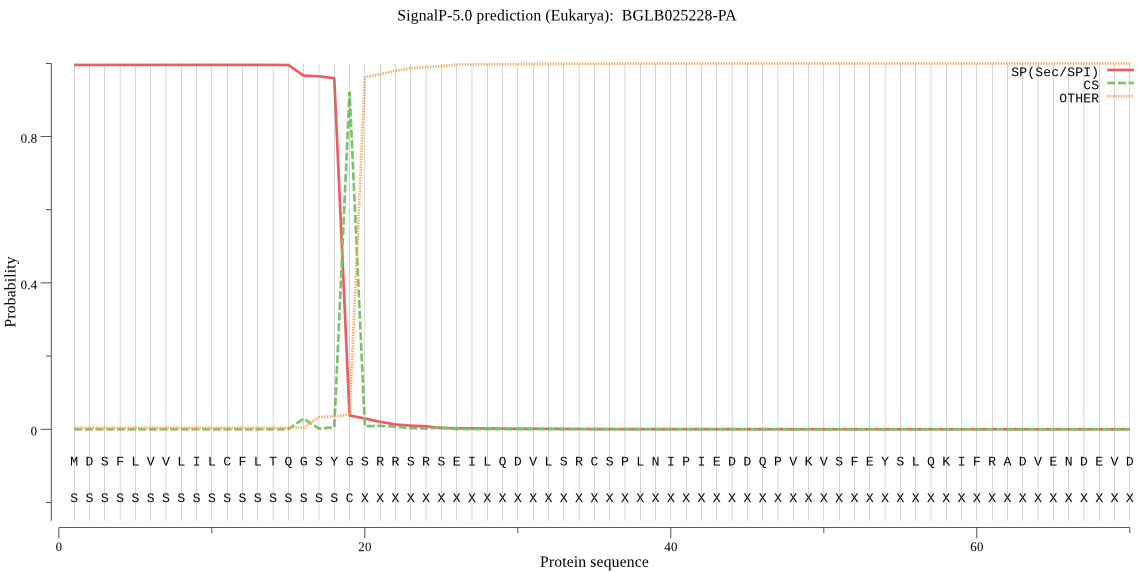


Description: Uncharacterized protein LOC106067104 isoform X1


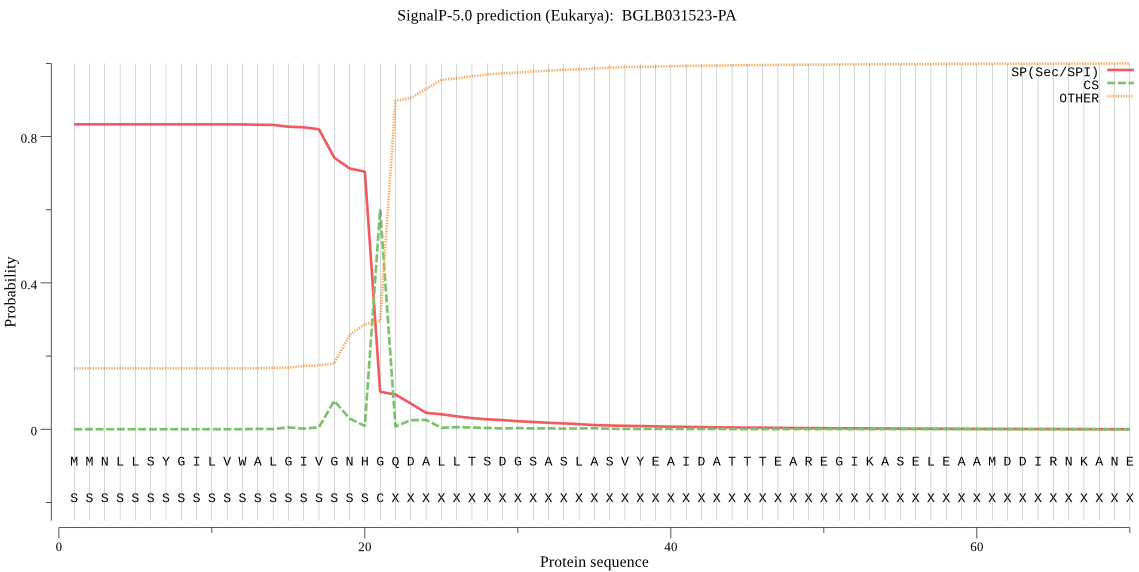

Supplement: File S1 — SignalP schematic of attractant candidates. [file Table_1.docx]
