# Supplementary material for: Identification of Schistosoma mansoni miracidia attractant candidates in infected Biomphalaria glabrata using behaviour-guided comparative proteomics
Source: Front Immunol. 2022 Oct 10;13:954282. doi: 10.3389/fimmu.2022.954282 (PMC9589101; doi:10.3389/fimmu.2022.954282)
Supplement: File S2 — Annotation of attractant candidates derived from B. glabrata SCW, including signal peptide (yellow highlight), dibasic cleavage sites (large bold), cysteine residue (red font) and amidation (underline). [file Table_2.docx]

> BGLB017354-PA (Uncharacterized protein LOC106070463)

MASTLLYNILICALGVVAGQGEQTVSIPVGLPDVAPIANLIDQTAAKAKEGVDAAELRTAMNSIERLADAYLDARDAKVDLQEIVNLLSELSTQFNYAIALSTFLEDPLVQEGRKVSVGLYILTE**C**SRLMTLSDNETMTNIIMATTSFTADAVSRGILNAEGIYIYRKEDLGNSKSPS**KR**WEVTGNVSKGPGGTTWSVSVSHKFG**KR**LTQRGEWSAGVNVGYSQPGGWSVGGSLSYKWG

> BGLB020983-PA/BGLB020983-PB (Acetylcholine-binding protein-like)

MDSLKVVLIVCLLIYGSHGTKKSREEIVKEILGRANPNNIPIVDEQPVKVSFKYSLQDIYTADVGTDQVELGLWLVISWKDRSLSWSNECTTFNELTLPSKYIWLPHIEVYNSIGKPGIHSDQLVRVYKDGTVTFVPQYTIRFSCALENVTTEQGAACTLKFGPWTYDVRDLVLDESQQVDLTTYAGGERFQLIEAKQKVNKKTYPCCPQSFEDIELRVTFKKI

> BGLB021783-PA (Uncharacterized protein LOC106056935)

MRIGSLLMVLTLGTCLSAESSPTTGHESESLGSNSRD**KR**LLFSGSVNLGGSLLGGLLDEKGLLDQLLRDTGLKTIVSGVLNVNGTLAQLLGGPDGLIGYIVKIVNDIFKTLDQTSQKSIVSGLARSPMLRNILSHVTSPNGLLASVGDVLNVVS**C**VL**KR**LHLDLGPVLDLLKAVTHLEQISVLKLG**C**NSTAGADLIGGLLGAKGGLTSLLDNLV**C**TVGDVVNGVFTSLQLQVAKGLLLSLPGFE**C**SNGTILNGTTSLLGLTSLVDSVFGGLLSNNQMVSLVTNILKNPSFLSGVLSGDVNFLKNILQAVV**C**LLNKLGFGDILKLVSNTLTPKQLLNLDLI**C**VNSSNDLLSTILGDKNLLNNIPLLTEIA**C**LVNKGIPVKDILSLTGPTNLLTFTEGA**C**EAGLLTNLLSNVSAFVIPLIQELQKINITLDQLNLEKVIVLVNALGLFGGDFVTFVAELT**C**FVKKLGLSLVTDIIRLIGIDKMLQFSTYV**C**KNGQPLFDEFDAPTQAKFVTAMGIFNVSDLTDIYNALK**C**LAEKLSWSTVLDFAIAVRSLDPLR**C**EQVTVGSLVGLIAGLVDNLGVDVVNYLLAQINPLVLLDLAKIFYINGSFNTTLFEKVN**C**MLAIIIIKLNAGELITSQADIEVLSSML**C**PNGTAINFPENLQQYLDVLRL**C**HNGLTAE**C**QSIMTATINKDS**C**KA**C**MTNDF**C**NGVNTYAPKEAATE**C**YAE**C**VA**C**TQATG

> BGLB025228-PA/BGLB025228-PB (Acetylcholine-binding protein-like)

MDSFLVVLILCFLTQGSYGSRRSRSEILQDVLSR**C**SPLNIPIEDDQPVKVSFEYSLQRIFRADVENDEVDIGLWTTLVWKDR**C**LNWFNEFTSFKELTVPIAEIWTPDIFLFDSVGAPEIFSDKLARVSQDGTVTYVPQLKVRLS**C**PLADLKLETGVT**C**SLKSGSWTHSTQELTLEVNAKVDLGDYASDTRFQLLNATQQVNRKQYP**CC**PETYDDATLYFTFRKP

>BGLB029661-PA (Uncharacterized protein LOC106080255)

MADFSLLFVLTLSVAVPRGNGELATLKFEAQPGTIHPILTKELELR**C**SVHNSNWKFEKSTTTTSFLQYFTTKQPLWNDTAKSNDTSNVQSSNSQDQAEFSRLLSLVVTKVNNESGENETIASVTG**C**DTPVIENRFVNTLQVVGSTEGSPVFGEQGYLTLTWDSPVEKDAGYFI**C**EAYALNSNKHPVSLSASLQINALQPQISDLVSYISTNDKYILDMKAKVTEVYEENQKLREQARELAFQNDYILNRLDQLRGQNSQNGRFQ**C**SYSTYIDFNPPFNSTPRVILSLASFSVSYSYSYSVSLQSVSNTSFSVF**C**SNPSGSTQVDWFATD

>BGLB031523-PA (Uncharacterized protein LOC106067104 isoform X1)

MMNLLSYGILVWALGIVGNHGQDALLTSDGSASLASVYEAIDATTTEAREGIKASELEAAMDDIRNKANEYLNGKSAKVERQDIVNLLADVTSKYNQGIALSTLLEDPLVQEDRKVSAGLYTLNELSRRLKKEGNETSINTLVASVQFTAGAIRGMVLTVESIYTYNMDVSNNENNSN**KR**LTKPGPFDGGYRGPYWAEGVRRQNVLRRLS**KR**PWSAGLSGGYSQGGGFNVGASVSYSWR
